# Periorbital Reconstruction by “Periorbital Patch” Technique Using a Pericardium-Based Collagen Membrane and Titanium Mesh

**DOI:** 10.3390/ma12152343

**Published:** 2019-07-24

**Authors:** Nenad Tanaskovic, Branko Trajkovski, Željka Perić Kačarević, Patrick M. Rider, Alireza Houshmand, Xin Xiong, Ole Jung, Mike Barbeck

**Affiliations:** 1 Clinic of Maxillofacial Surgery, Clinical Centre, 78000 Banja Luka, Herzegovina; 2 Wound Healing and Oral Diagnostic Research Group, College of Dental Medicine, University of Sharjah, 27272 Sharja, UAE; 3 Botiss Biomaterials GmbH, 15806 Zossen, Germany; 4 Department of Anatomy Histology, Embryology, Pathology Anatomy and Pathology Histology, Faculty of Dental Medicine and Health, University of Osijek, 31000 Osijek, Croatia; 5 Natural and Medical Sciences Institute, University of Tübingen, 72770 Reutlingen, Germany; 6 Department of Oral and Maxillofacial Surgery, Division for Regenerative Orofacial Medicine, University Medical Center Hamburg-Eppendorf, 20251 Hamburg, Germany; 7 BerlinAnalytix GmbH, 12109 Berlin, Germany

**Keywords:** orbital adherence syndrome, titanium mesh, fibrosis, periosteum, collagen membrane

## Abstract

Objective: Titanium mesh is a commonly used material for the reconstruction of orbital floor fractures. However, in some instances, a subsequent inflammatory reaction can occur that causes the adhesion of orbital tissue to the titanium mesh. The adhesion of the orbital soft tissue to the mesh causes diplopia, lid rigidity and extraocular movements restriction. This study was performed to determine if the placement of a collagen membrane over a titanium mesh can prevent the adhesion of orbital soft tissue for an improved clinical outcome. Clinical considerations: A case study was performed investigating 106 patients undergoing a periorbital restoration. Seventy-two patients received a titanium mesh without a barrier membrane, 12 patients received a barrier membrane composed of autologous auricular cartilage to provide a barrier function and 22 patients received a pericardium collagen membrane and titanium mesh. Conclusions: Titanium has been shown to generate an intense inflammatory reaction in host tissues, which can cause fibrosis to adjacent structures. Fibrosis is an essential factor in the repair of fracture sites, however this can lead to adverse effects in the orbital socket. Fibrosis can cause cicatrization and lower eyelid retraction when induced along the lower orbital rim. An improved outcome can be achieved by using a barrier between the titanium mesh and the soft tissue, such as autogenous auricular cartilage, however, only patients treated with a resorbable collagen membrane to act as a soft tissue barricade during site regeneration, prevented the fibrosis reaction and related problems from occurring.

## 1. Introduction

Orbital floor fractures can occur following blunt force trauma to the eye socket, most commonly caused by sports injuries, traffic accidents and physical assault. The orbit can become damaged due to a buildup of pressure inside the orbital socket that forces the orbital contents through the medial and inferior walls. The extent of damage depends upon the severity and/or localization of the injury, and can affect the bulbus oculi, the eye muscles, as well as nerves located within the socket [1]. Treatment is required to prevent avoidable complications such as diplopia, enophthalmos, paresthesia, extraocular muscle movement limitations and retraction of the eyelids. Even after treatment, some of these symptoms can persist, for example, persistence of diplopia and movement limitation after surgical interventions are reported in up to 20% of the treated cases [2,3]. These issues can be caused by muscle or periorbital soft tissue entrapment in the fracture line, muscle injury or soft tissue adherence to the supporting periorbital implant [4].

Materials used for reconstructive surgery are varied and their use is largely dependent upon the surgeon’s preference and the chosen technique. Autologous tissues such as bone, temporalis fascia and cartilage are most commonly used as they do not present the risk of rejection and are able to provide adequate support. However, using autologous material increases morbidity for the patient as a second surgical site is required for tissue extraction. It has been reported in some cases that the donor site can cause a higher level of pain than that of the augmentation site [5]. The use of autologous material also increases surgical time, has unpredictable resorption and is difficult to model to an appropriate shape.

An ideal material should restore volume and the anatomic form of the area, provide easy placement, and produce minimal/no inflammatory reaction. A range of materials can be used to repair orbital floor fractures depending upon their size. While both autologous transplants, allogeneic and most synthetic materials have shown to be suitable for treatment of orbital wall fractures, most material types are not often clinically applied due to the associated complications [6].

Titanium has been demonstrated as an excellent repair material for orbital floor restorations [7,8]. It has a high strength to weight ratio that provides mechanical support and a resilience to deformation and dislocation over time. It is biocompatible, provoking a minimal foreign body reaction and has excellent osseointegration. Titanium does not corrode within the body, therefore its mechanical properties do not change and it does not release corrosion by-products into the surrounding tissues.

Although the reported success rates are relatively high in cases using titanium meshes to repair orbital floor fractures, eye motility, eyelid mal-positioning and retraction can remain after surgery [4,9,10]. In large sized orbital defects, the titanium implant will be in contact with both the bony structural aspect of the orbit as well as the moving soft tissues. Due to a high porosity and high surface tension of the commercially available titanium meshes, the soft tissues can easily adhere to the implant [4]. The interaction of the soft tissue with the titanium can induce an inflammatory reaction that can further provoke the adhesion of the orbital contents on to the titanium [10]. The sharp edges of the titanium mesh can perforate the soft tissue, thereby increasing its contact and integration with the soft tissue leading to increased adhesion [10]. Adhesion of the orbital contents to the titanium mesh can also be caused by a fibrotic tissue formation as part of a foreign body reaction [11]. The fibrosis anchors the soft tissues to the orbital rim and causes a serious pathologic process such as orbital adherence syndrome. This process is referred to as orbital adherence syndrome and is usually manifested with reduced eye motility as well as eyelid mal-positioning and retraction [9].

Different methods have been applied to separate the soft tissue from the titanium mesh and thereby reduce the risk of orbital adherence. Chang and Lee (2014) used a silicone membrane during surgery to separate the soft tissue from the titanium mesh. By using the silicone barrier membrane, they were able to reduce the contact of the herniated tissue contents and reduce the rate of complications such as diplopia or enophthalmos [12]. Other studies have been performed that investigate the use of partially resorbable titanium meshes. These are titanium meshes that are usually embedded in an synthetic polymer matrix [13,14]. The polymer acts as a barrier between the titanium and the orbital soft tissues, however, the degradation products of synthetic biodegradable polymers can cause problems if they accumulate in the surrounding tissues, such as an increase in an inflammatory reaction.

The use of the maxillary anterior wall of the sinus, septal and auricular cartilage have also been applied for the repair of orbital fractures and can be used in conjunction with the titanium mesh to provide a barrier to the titanium. However, these autologous materials are limited by the amount of material that can be sourced, hence the size of the defect that can be treated. As the material is autologously sourced, a second surgical site is required, increasing surgical times. Therefore, a more convenient alternative would be to use a graft material that is not sourced from the patient’s own body. An alternative to the use of partially resorbable titanium meshes or using autologous material to provide a barrier function to the titanium mesh, could be to use a collagen barrier membrane in combination with the titanium mesh. Collagen membranes are well established for bone augmentation procedures, specifically in oral surgeries, where they are used to separate the hard and soft tissues during bone regeneration [15,16]. Collagen membranes can provide a controlled degradation rate and degrades into components that are recycled by the body, making it an ideal material for its application.

In this paper we present a “periorbital patch” technique that allows grafting of fat and perifascial areolar tissue by means of titanium meshes in a combination with a commercial pericardium-based collagen barrier membrane (Jason^®^ Membrane, Botiss Biomaterials GmbH, Zossen, Germany), originally intended for guided bone regeneration in oral surgeries. The Jason^®^ Membrane has been used successfully to secluded epithelial and connective tissues from defect site, allowing for the proper regeneration of bone in defect sites [15,16]. It was hypothesized that the same barrier principle provided by the collagen membrane could enable periosteal cell migration to repair the periorbital structures during the postoperative healing period, whilst avoiding adhesion of these structures to the titanium mesh surface, thereby reducing the rates of post-operative instances of lid retraction, diplopia and restricted extraocular movements. In this context, the “periorbital patch” technique with a collagen membrane was applied in 22 patients with orbital wall fractures and retrospectively observed in regard to eye movements and lid retraction. These results were compared to those of 72 patients treated with a titanium mesh only, and 12 patients treated with the titanium mesh and autologous auricular cartilage to provide a barrier function. 

The null hypothesis of this study is that at six months post-surgery, patients treated with the titanium mesh and collagen membrane will have the same rate of occurrences of diplopia, lid rigidity and extraocular movement limitations as those patients treated only with a titanium mesh.

## 2. Method

### 2.1. Patients

This study involved 106 patients with orbital floor fracture that were referred to our hospital during the period of December 2011 and July 2015. All subjects gave their informed consent for inclusion before they participated in the study. The study was conducted in accordance with the Declaration of Helsinki, and the protocol was approved by the Ethics Committee of the Clinical Center Banja Luka (01-8727-2/10).

In the primary position, up or down gaze and right or left gaze, the patients were examined for binocular diplopia. Reconstructions were performed by a single surgeon (N.T., Nenad Tanaskovic). During this period, reconstructive surgeries were performed using either the standardize titanium mesh approach, a titanium mesh with autologous auricular cartilage, or a titanium mesh with a xenogeneic collagen membrane.

Seventy-two patients were treated using only a titanium mesh for orbital wall reconstruction. This patient group consisted of fifty-five men and seventeen women that had an average age of 38 years. 12 reconstructive surgeries were performed using an auricular cartilage. This series of patients included nine males and three females, with an average age of 35 years. Cartilage grafts were placed on the healthy orbital edge, without being fixated to the base. 22 patients were treated with a titanium mesh combined with a collagen membrane; twenty males and two females that had an average age of 37 years.

The presence of binocular diplopia was objectively evaluated by elimination of diplopia after closing one eye. Preoperatively, diplopia was noted in 73 cases (69%) and a limitation of extraocular movement in 55 cases (51%). 

Computer tomography (CT) and orthoptic examinations were performed on all patients on their first visit and one day after surgery. The orthoptic examinations included the Hess screen test (Electronic Hess Screen; Haag-Streit Holdings AG, Clement Clarke International Ltd., Essex, UK). The Hess screen test determines the extent of eye movements preoperatively using Hess chart examinations. Axial, coronal and sagittal multislice continuous CT scans were made with a 1 mm section thickness. Limitation of ductions in horizontal and vertical gazes was measured using a five-point scale (0 to −4), where “0” represented no limitation and “−4” represented no movement beyond the midline.

After complete clinical and physical examinations, all surgeries were performed by one surgeon, mostly via the subciliary and lateroorbital approach, and in some cases bicoronal and glabellar approaches.

Seventy-two patients received a titanium orbital mesh plate (Synthes CMF, Oberdorf, Switzerland); 22 patients received a combination of a pericardium-based collagen membrane (Jason^®^ Membrane, Botiss Biomaterials GmbH, Berlin, Germany) and a titanium orbital mesh plate and 12 patients received a reconstruction of the orbital wall using a 1.5 cm wide titanium ribbon on which a cartilage graft of the left auricle was added in the deep region of the orbit. Forced duction tests were performed under general anesthesia at the beginning and end of the operation. The average length of hospitalization was two days with an average follow up period of 15 months.

Statistical analysis was performed using the statistical software GraphPad Prism (GraphPad Prism version 8; GraphPad Software Inc., San Diego, CA, USA). The statistical significance was calculated using contingency tables with a Fisher’s exact test, which took into account the large variation in number of patients between groups. The differences were considered significant if *p*-values were less than 0.05 (* *p* < 0.05).

### 2.2. Surgical Technique

A subciliary incision was used to access the orbital floor fractures. Orbital septum incisions were used to directly approach adhesions between the extraocular muscle and periorbita. The orbital septum was incised from the level of the caruncle until the lateral canthus and just above the orbital rim. Therefore, the inferior rectus muscle, inferior oblique muscle and the medial rectus muscle were usually exposed during this process. Subsequently, bipolar electrocautery and scissors were used to separate and remove the orbital fat and muscle adhered to the orbital margin and the orbital floor.

**Figure 1:**
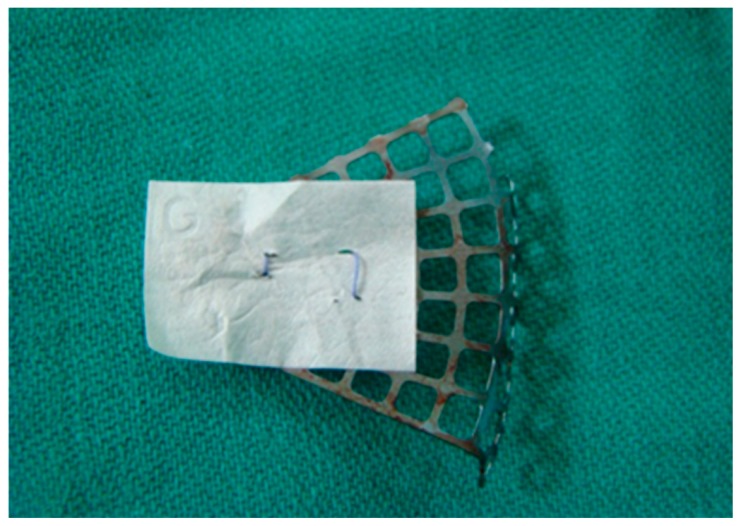
Collagen membrane partially covering the titanium orbital mesh plate and fixed via sutures.

For the first four patients treated with a collagen membrane in combination with the titanium mesh, this tissue was grafted over the ruptured periorbita with fixation to the collagen membrane that was partially covering the titanium mesh (Figure 1). In a six-month follow-up examination of these four patients, normal and symmetrical eye movements with the presence of eyelids rigidity were observed. Therefore, the treatment of the following eighteen patients was modified so that the titanium mesh was completely covered by the collagen membrane.

**Figure 2:**
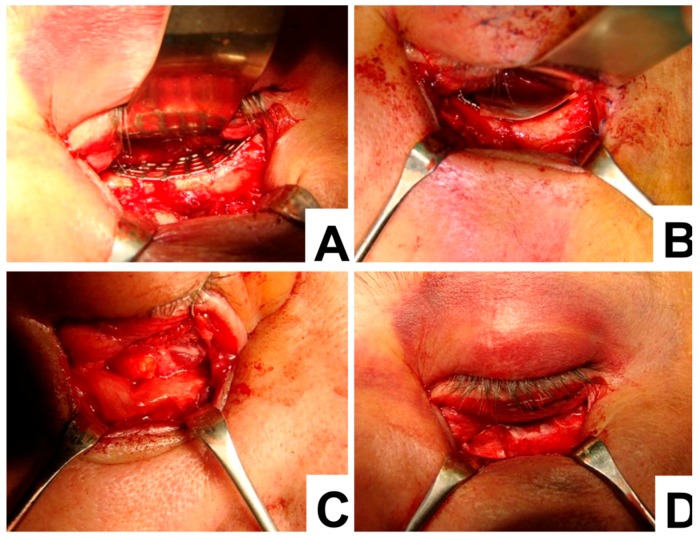
Surgical technique. (**A**) Titanium mesh set parallel to the orbital frame; (**B**) collagen membrane set parallel to the titanium mesh edge; (**C**) prolapsed orbital fat and collagen membrane and (**D**) titanium mesh, fully covered with collagen membrane.

An example of the surgical procedure involving the titanium mesh and collagen membrane is demonstrated below (Figure 2). To begin with, the titanium mesh was placed over the orbital floor (Figure 2A). Then the collagen membrane was placed to the edge of the titanium mesh (Figure 2B). This caused the prolapsed orbital fat and collagen membrane to completely cover the titanium mesh, the orbital floor and the orbital rim (Figure 2C,D). Once the membrane was positioned, a forced duction test was used to check the patient’s eye movement. When eye movement was not restricted, the orbital septum and skin were closed.

## 3. Results

22 patients underwent combined reconstruction of the orbital floor using both a titanium mesh and a collagen membrane. In the first four patients, the membrane was stitched to the titanium mesh in order to cover the defect at the point of rupture as the anterior part of the mesh remained uncovered (Figure 1). Although postoperatively these patients had no limitation to their ocular movements, there was a retraction of the lower eyelid. The retraction of the eyelid might be caused by tissue adhesion to the mesh in the orbital rim region. For that reason, in the following two patients the membrane was positioned parallel to the anterior edge of the mesh, however this too resulted with the same outcome as for the four previous patients.

**Figure 3:**
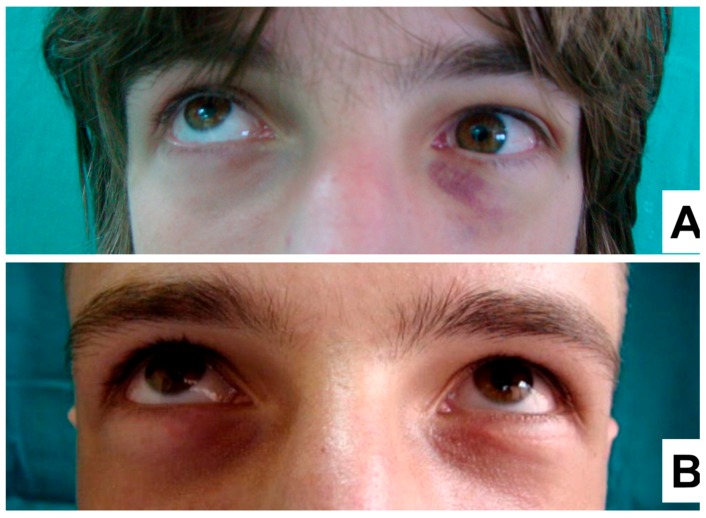
Pre- (**A**) and postoperative (**B**) situation of the eyelids in the patient undergoing orbital floor fracture treatment by a fully covered titanium mesh with collagen membrane. (**A**) Preoperative up-gaze restriction, as a result of the fracture of the left orbital floor. (**B**) Eye movements without any restrictions and symmetrical height of the eyelids without one sign of rigidity and cicatrization. Significantly improved extent of the eye movements and lack of lid retraction was observed.

Therefore, for all the subsequent patients, a fully covered titanium mesh was used for the orbital floor reconstruction method. In these patients, a significant improvement to the extent of the eye movements was observed, as well as a reduction in the adhesion of orbital soft tissue, late postoperative diplopia, up gaze movement restriction and lack of lid retraction (Figure 3A,B).

**Figure 4:**
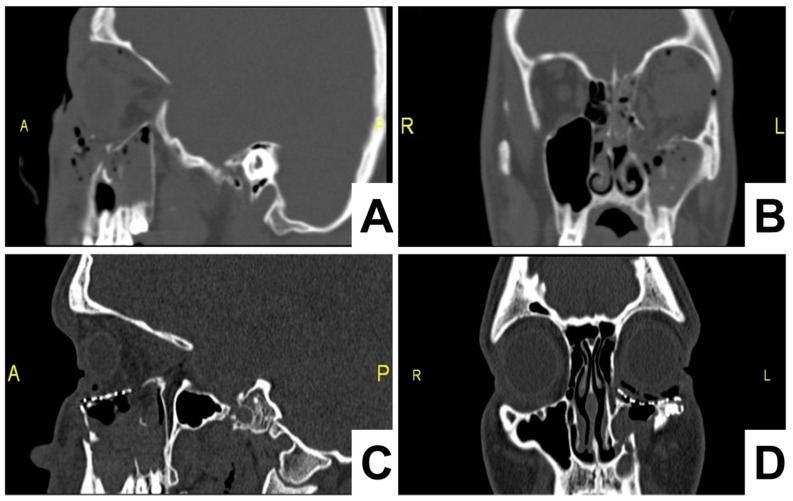
Pre- (**A**,**B**) and postoperative (next day) (**C**,**D**) control computer tomography (CT) scan of the eyelids in the patient undergoing orbital floor fracture treatment by a fully covered titanium mesh with collagen membrane. A benefit of the titanium mesh is that it does not present artifacts in CT or magneitc resonance (MR, Magnetic resonance) imaging.

On the first postoperative day, a control CT scan was performed to assess the location of the implant. The postoperative follow-up was performed at days 7 and 14 days, which confirmed a favorable outcome, without restriction in the movements of the eyeball and corresponding height of the lower eyelid (Figure 4).

Eye motility was measured preoperatively and postoperatively at every follow-up appointment (Table 1, Table 2 and Table 3). Both the titanium mesh and the titanium mesh with collagen produced a significant increase in the number of patients without eye motility issues after six months. In comparison, for patients treated with the titanium mesh and cartilage graft, there was no significant change to the number of patients within each motility ranking over the course of the study.

The summary findings of diplopia, lid rigidity and extraocular movement limitations in all the patients are shown in Table 4, Table 5 and Table 6. The use of a barrier between the titanium mesh and the soft tissue was shown to be beneficial after six months. Although the occurrences of diplopia, extraocular movements (EOM) limitation and rigidity of lids were not present at one month in the titanium mesh group (Table 4), after six months, this group exhibited the highest rate of occurrences. Whereas the use of titanium mesh in combination with the collagen barrier membrane recorded no occurrences of diplopia, EOM limitation and rigidity of lids (Table 6).

**Figure 5:**
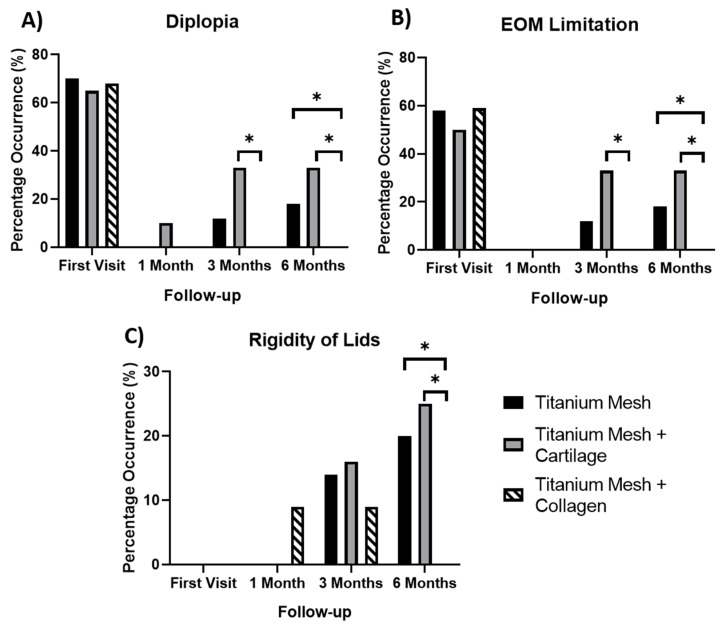
Percentage of occurrence of (**A**) diplopia; (**B**) EOM limitations and (**C**) rigidity of lids, for each time point of the study (* *p* < 0.05).

The significance of diplopia, lid rigidity and EOM limitation results are demonstrated in Figure 5. The use of a collagen membrane in combination with the titanium mesh is shown to be beneficial six months post-surgery. At six months, instances of diplopia, EOM limitation and rigidity of lids were significantly higher in patients treated the titanium mesh alone in comparison to those treated with a titanium mesh and a collagen membrane (*p* values of 0.0341, 0.0341 and 0.0349 respectively). Instances of diplopia, lid rigidity and EOM limitations were significantly higher at three months and six months in patients treated using cartilage as a barrier instead of collagen.

## 4. Discussion

The orbital floor reconstruction is clinically used for repositioning of the herniated orbital contents, reconstruction of the orbital floor defect and achievement of the anatomically correct orbital volume. In this context, titanium meshes are most often used for reconstruction of the orbital floor as it has generally been shown that titanium is useful for the treatment of comminuted fractures in order to bridge over multiple bone fragments [4,17]. Moreover, most orbital wall fractures are often associated with defects or dislocations of periorbital structures, which include the periosteum, the Lockwood ligament and the periorbital fat that act as a protective layer between the globe, extraocular muscles and the bony orbit, enabling a smooth gliding interface [18,19,20]. Small fat components may become exposed to the orbital mesh in instances of periorbital disruption due to fracture of the orbital floor. Fat can penetrate the titanium mesh pores and entwine with the mesh due to swelling. This can further result in muscle tethering and clinically manifest as a gaze restriction in the early and late postoperative period [1,21]. Finally, a fat necrosis can occur that causes an inflammatory response leading to worsening of the symptoms over time.

It is well known that increased surface irregularities and roughness of titanium implants result in a larger bone-implant interfacial area, resulting in enhanced osseointegration and a stronger host bone to implant adhesion [22]. However, the osseointegration itself is regarded as an immune-modulated inflammatory process that can cause a fibrotic reaction towards the titanium mesh implant [4,11,23]. The use of titanium orbital floor implants anchored to the infraorbital rim can also result in greater surface irregularities and orbit-eyelid exposure to the titanium mesh that can cause an increased fibrosis reaction [24].

A fibrotic response towards to the commonly used titanium meshes can result in orbital adherence syndrome. This is caused by fibrous tissue ingrowth through the pores of the mesh. As a result, periorbita tethering, hindrance of upward globe movements and cicatricial eyelid retraction may occur. The “periorbital patch” technique using a commercial collagen barrier membrane has been proposed as a possible solution to overcome this clinical issue, and prevent fibrous tissue attachment and ingrowth into the titanium mesh. It has already been shown that collagen membranes are optimally suitable as barrier materials by preventing the fast invasion of cells, by separating soft and hard tissue in alveolar ridge augmentations and prevention of the epithelium migration in guided bone regeneration [25].

The long-term degradation (3–6 months) of the pericardium-based collagen membrane, when used in combination with a titanium mesh, can provide a sufficient barrier function in periorbital cleft coverage, thereby supporting periosteal tissue creeping and defect closing [26]. At a six-month post-operative checkup, it is shown that eye movements were significantly improved by using a collagen barrier membrane in combination with titanium mesh for orbital floor reconstruction.

The extent of eye movement was analyzed for 106 patients treated with either a titanium mesh, a titanium mesh with autologous auricular cartilage or a titanium mesh with a collagen membrane. Results obtained using a five-point scale to measure limitation of duction in horizontal and vertical gazes, as well as by recording the frequency of diplopia, lid rigidity and extraocular movements (EOM) limitations, demonstrated a distinct improvement after application of the collagen membrane. The combined use of a collagen barrier membrane with the titanium mesh provided results that were significantly better than that of the titanium mesh alone after six months. Therefore, it has been demonstrated that the same principle used in Guided Bone Regeneration (GBR) applications can be successfully applied to periorbital regeneration and improve post-operative outcomes. However, the combined use of a cartilage graft and titanium mesh proved to be less effective as the collagen membrane at providing the barrier function. The proposed technique enabled bone to be regenerated via the stability and volume provision provided by the titanium mesh, whilst the use of a collagen membrane prevented dislocations of the periorbital tissue. The long-term barrier function can eliminate the risk of periorbital fat prolapse and adhesion to the titanium mesh. In comparison to the use of autologous cartilage, collagen presents an off-the-shelf alternative, reducing surgical times and morbidity for the patient, whilst providing the same barrier function.

Moreover, it is possible that periorbital structures like the periosteum and the Lockwood ligament have successfully been repaired based upon the results of the EOM, as the function of these structures are important for eye movements and provide a gliding interface. In this context, the collagen might not function purely as a barrier material, but also as an extracellular matrix, supporting the periosteal tissue. The periosteum, which is a soft tissue layer of bone tissue, consisting of the *Stratum fibrosum* and the *Stratum osteogenicum*, are already physiologically composed of collagen [26]. Furthermore, collagen-based biomaterials have been used for successful regeneration and repair of ligament tissue [27,28]. Altogether, the “periorbital patch” technique using a collagen barrier membrane might be a suitable strategy for repair of periorbital structures during the postoperative healing period while avoiding adhesion of these structures to the titanium mesh surface, possible fibrosis and post-operative complications. However, further preclinical and clinical studies have to prove these findings.

## 5. Conclusions

The periorbital reconstruction treatment can lead to several post-operative complications. An alternative “periorbital patch” technique, combining the use of a titanium mesh and a collagen membrane has resulted in no post-operative symptoms, not observed in the surgeries performed with only a titanium mesh, or with a titanium mesh in combination with autologous auricular cartilage. Restrictions to the eye movements were greatly improved. It is recommended to further evaluate and monitor more cases in order to conclude if this technique leads to statistically significant long-term results.

## Figures and Tables

**Table 1:** Vertical and horizontal five-point scale (0 to −4) for 72 titanium meshes.

Motility	First Visit	1 Month	3 Months	6 Months	Statistical Significance
0	30	72	63	59	****
−1	9	0	3	4	ns
−2	13	0	2	3	*
−3	12	0	2	3	*
−4	8	0	2	3	ns

Motility ranked as: 0 = normal motility; −1 = mild restriction; −2 = moderate; −3 = severe; −4 = no motility. Statistical significance calculated between the first visit and the six-month follow-up (ns = *p* > 0.05, * = *p* < 0.05, **** = *p* < 0.0001).

**Table 2:** Vertical and horizontal five-point scale (0 to −4) for 12 patients treated with ear cartilage.

Motility	First Visit	1 Month	3 Months	6 Months	Statistical Significance
0	6	12	8	8	ns
−1	4	0	0	0	ns
−2	0	0	2	2	ns
−3	1	0	2	2	ns
−4	1	0	0	0	ns

Motility ranked as: 0 = normal motility; −1 = mild restriction; −2 = moderate; −3 = severe; −4 = no motility. Statistical significance calculated between the first visit and the six-month follow-up (ns = *p* > 0.05).

**Table 3:** Vertical and horizontal five-point scale (0 to −4) for 22 patients treated with collagen membrane.

Motility	First Visit	1 Month	3 Months	6 Months	Statistical Significance
0	9	22	22	22	****
−1	5	0	0	0	*
−2	4	0	0	0	ns
−3	2	0	0	0	ns
−4	2	0	0	0	ns

Motility ranked as: 0 = normal motility; −1 = mild restriction; −2 = moderate; −3 = severe; −4 = no motility. Statistical significance calculated between the first visit and the six-month follow-up (ns = *p* > 0.05, * = *p* < 0.05, **** = *p* < 0.0001).

**Table 4:** The frequency of diplopia, lid rigidity and extraocular movements (EOM) restriction for patients treated only with a titanium mesh (72 patients).

Analysis Parameter	First Visit	1 Month	3 Months	6 Months
Diplopia	70%	None	12%	18%
EOM Limitation	58%	None	12%	18%
Rigidity of Lids	None	None	14%	20%

**Table 5:** The frequency of diplopia, lid rigidity and extraocular movements (EOM) restriction for patients treated with a titanium mesh covered with cartilage from the ear (12 patients).

Analysis Parameter	First Visit	1 Month	3 Months	6 Months
Diplopia	65%	10%	33%	33%
EOM Limitation	50%	None	33%	33%
Rigidity of Lids	None	None	16%	25%

**Table 6:** The frequency of diplopia, lid rigidity and extraocular movements (EOM) restriction of patients treated with the titanium mesh and a collagen membrane (22 patients).

Analysis Parameter	First Visit	1 Month	3 Months	6 Months
Diplopia	68%	None	None	None
EOM Limitation	59%	None	None	None
Rigidity of Lids	None	9%	9%	None
